# The effects of rTMS on self-reported quality of life in younger and older adults with major depressive disorder

**DOI:** 10.1017/S0033291725102079

**Published:** 2025-10-08

**Authors:** Katharina Göke, Jonathan Downar, Fidel Vila-Rodriguez, Zafiris J. Daskalakis, Tarek K. Rajji, Benoit H. Mulsant, Daniel M. Blumberger

**Affiliations:** 1 Temerty Centre for Therapeutic Brain Intervention and Campbell Family Research Institute, Centre for Addiction and Mental Health, Toronto, ON, Canada; 2 Institute of Medical Science, University of Toronto, Toronto, ON, Canada; 3 Department of Psychiatry, Temerty Faculty of Medicine, University of Toronto, Toronto, ON, Canada; 4 Non-Invasive Neurostimulation Therapies Laboratory, Department of Psychiatry, Faculty of Medicine, University of British Columbia, Vancouver, BC, Canada; 5 School of Biomedical Engineering, Faculty of Applied Science | Faculty of Medicine, University of British Columbia, Vancouver, BC, Canada; 6 Department of Psychiatry, University of California, San Diego Health, La Jolla, CA, USA; 7 Department of Psychiatry, University of Texas Southwestern Medical Center, Dallas, TX, USA

**Keywords:** late-life depression, quality of life, repetitive transcranial magnetic stimulation, theta burst stimulation, treatment-resistant depression

## Abstract

**Background:**

Repetitive transcranial magnetic stimulation (rTMS) is a well-established intervention for treatment-resistant depression. However, its effects on patient-reported outcomes, such as quality of life (QoL), have not been fully characterized, especially among older adults. This study compares the impact of rTMS on QoL in younger (<60 years) versus older (≥60 years) adults with major depressive disorder.

**Methods:**

We analyzed data from 531 participants with depression (ages 18–89 years) from two randomized clinical trials (THREE-D and FOUR-D). All participants received either unilateral or bilateral rTMS or theta burst stimulation. QoL was assessed using the Quality of Life Enjoyment and Satisfaction Questionnaire – Short Form at baseline, end of treatment, and 12-week follow-up, and compared between younger adults (age < 60 years; *n* = 360) and older adults (age ≥ 60 years, *n* = 171). The clinical relevance of the changes was evaluated through effect sizes, using a predefined threshold of 12 points as the minimal clinically important difference, and comparisons with community norms.

**Results:**

After rTMS treatment, both younger and older adults experienced statistically significant improvements in QoL, with medium to large effect sizes. The effect was sustained over 12 weeks of follow-up. At baseline, only 0.3% of younger adults and 2.3% of older adults reported normal QoL, which significantly increased to, respectively, 19.8 and 19.4% by the end of treatment, and 23.7 and 26.8% at the 12-week follow-up.

**Conclusions:**

rTMS yielded acute and sustained clinically meaningful improvements in QoL, with similar effects among younger and older adults with depression. The magnitude of improvement was comparable to, or exceeded, that reported in antidepressant trials.

## Introduction

Major depressive disorder (MDD) is a leading contributor to the global burden of disease, impacting around 300 million adults across all age groups (GBD 2019 Diseases and Injuries Collaborators, 2020; Herrman et al., 2019; World Health Organization, 2023). Beyond its clinical symptoms, MDD severely impacts an individual’s quality of life (QoL), which encompasses their subjective evaluation of their psychological, social, and physical well-being (IsHak et al., 2011). Despite the availability of pharmacotherapy and psychotherapy, 30–40% of patients with MDD develop treatment-resistant depression (TRD), a condition in which patients do not achieve adequate symptom relief even after multiple treatment attempts (Gaynes et al., 2020). TRD not only leads to increased disability rates, higher medical costs, and worse long-term outcomes but also contributes to further declines in QoL (Fekadu et al., 2009; Johnston et al., 2019; Mrazek et al., 2014; Rizvi et al., 2014).

Repetitive transcranial magnetic stimulation (rTMS) is a well-established treatment for TRD and involves the noninvasive delivery of repeated, powerful magnetic field pulses to the dorsolateral prefrontal cortex (DLPFC). Large-scale randomized controlled trials and meta-analyses confirmed its effectiveness for patients who do not respond to first-line therapies (Brunoni et al., 2017; O’Reardon et al., 2007; Sehatzadeh et al., 2019). While the effect of rTMS on depressive symptoms is well-documented, its impact on QoL remains less clear. Although depressive symptom severity typically correlates with QoL (Giacobbe et al., 2020; Hofmann et al., 2017; IsHak et al., 2011; Sivertsen et al., 2015), they explain only part of the variability in QoL (Cohen et al., 2013; Ishak et al., 2013; Rapaport et al., 2005; Trompenaars et al., 2006). Therefore, assessing QoL as a separate outcome measure in rTMS studies is essential to capture its broader effects on life satisfaction.

Age plays an important role in both the clinical presentation of and treatment response to MDD. MDD in older adults (≥60 years) poses unique clinical challenges, with higher treatment resistance, relapse rates, and recurrence rates (Knochel et al., 2015). Some early studies suggested that rTMS might be less effective in older adults than in younger individuals (Fregni et al., 2006; Manes et al., 2001; Mosimann et al., 2004). However, more recent studies have shown that older adults with MDD respond equally well (Ciobanu et al., 2013; Leuchter et al., 2024; Lisanby et al., 2009; Sackeim et al., 2020; Valiengo et al., 2022), or in some cases, even better than younger adults (Desbeaumes Jodoin et al., 2019; Fitzgerald et al., 2016).

Despite these advances, there remains a relative paucity of research exploring the effects of rTMS on QoL, particularly in older adults. Although a few studies have reported QoL improvements with rTMS in younger adults with MDD (Dumas et al., 2012; Giacobbe et al., 2020; Solvason et al., 2014), none have compared these outcomes between younger and older adults. In this context, the current analysis aimed to evaluate the effects of rTMS on QoL in both younger (<60 years) and older (≥60 years) adults with MDD, using data from two large rTMS randomized trials (THREE-D and FOUR-D). We hypothesized that rTMS would lead to significant improvements in QoL, with sustained effects over a follow-up period. We then compared these effects between younger and older adults with MDD. Additionally, we investigated the relationship between depressive symptoms and QoL improvement and examined QoL impairment relative to community norms.

## Methods

### Participants

This secondary analysis combined data from two randomized non-inferiority trials comparing standard rTMS versus theta burst stimulation (TBS). The first trial (THREE-D; ClinicalTrials.gov Identifier: NCT01887782; Blumberger et al., 2018) enrolled participants aged 18–65 years, while the second trial (FOUR-D; ClinicalTrials.gov Identifier: NCT02998580; Blumberger et al., 2022) enrolled participants 60 years of age and older. Both studies included outpatients with a primary diagnosis of MDD confirmed with the Mini-International Neuropsychiatric Interview (Sheehan et al., 1998). Other inclusion criteria included a 17-item Hamilton Rating Scale for Depression (HRSD-17; Hamilton, 1967) score of at least 18 (in the THREE-D trial) or a Montgomery–Åsberg Depression Rating Scale (MADRS; Montgomery & Asberg, 1979) score of at least 18 (in the FOUR-D trial). Moreover, participants had not responded to one or more antidepressant trials or they had not tolerated two or more antidepressants, and they had received stable dosages of antidepressant medications for at least 4 weeks before treatment. Further eligibility criteria were similar between the two trials and are detailed in the respective original articles (Blumberger et al., 2022, 2018). Both studies had been approved by local research ethics boards at all study sites, and all participants gave written, informed consent.

### rTMS treatment

In the THREE-D trial, participants were randomized to receive either high-frequency stimulation (10 Hz; 3,000 pulses per session) or intermittent TBS (iTBS; 600 pulses per session) to the left DLPFC. In the FOUR-D trial, participants were randomized to receive either bilateral standard rTMS (low frequency [1 Hz; 600 pulses per session] right-sided followed by high-frequency [10 Hz; 3000 pulses per session] left-sided) or bilateral TBS (right-sided continuous TBS [cTBS; 600 pulses per session], followed by left-sided iTBS [600 pulses per session]). In both trials, the stimulation target was 120% resting motor threshold, and treatment was delivered daily for 5 days per week for 20–30 days.

### Measures

Self-reported QoL was measured using the Quality of Life Enjoyment and Satisfaction Questionnaire – Short Form (Q-LES-Q-SF) (Endicott et al., 1993), collected at baseline, at the end of treatment, and 12 weeks post-treatment for both trials. The Q-LES-Q-SF comprises 14 items that assess satisfaction across various domains (e.g. work, leisure activities, family relationships, physical health, and others) on a 5-point Likert scale. Two additional items – satisfaction with medication and overall life satisfaction – are scored separately and not included in the total score. The 14 core items are summed to create a total QoL score (range: 14–70). This raw score is then converted into a percentage of the maximum possible score using the formula: (raw score − minimum possible score) / (maximum possible score − minimum possible score). The resulting percentage score ranges from 0 to 100, with higher percentages indicating greater enjoyment and satisfaction with life. The Q-LES-Q percentage score was used in all subsequent analyses and is referred to as ‘Q-LES-Q score’. To facilitate interpretation of this score, QoL was categorized based on previously defined cutoffs derived from a large community sample (Schechter et al., 2007). The average Q-LES-Q score in this community sample was 78.3 (standard deviation [SD] = 11.3) (Schechter et al., 2007). Scores within 1 SD below the norm (i.e. 67 or higher) are considered to reflect ‘within normal QoL’. Scores between 1 and 2 SDs below the norm (i.e. between 55.7 and 67) indicate ‘mild to moderate QoL impairment’, and scores 2 or more SDs below the norm (i.e. 55.7 or lower) indicate ‘severely impaired QoL’ (IsHak et al., 2024, 2018; Morton et al., 2021; Schechter et al., 2007; Steiner et al., 2017). To elucidate the clinical importance of a change on the Q-LES-Q, a minimal clinically important difference (MCID) of a 12-point change on the Q-LES-Q has been established in previous studies (Conway et al., 2018; Endicott et al., 2007; Rush et al., 2021).

Depressive symptom severity was measured using the Quick Inventory of Depressive Symptomatology 16-item self-report (Rush et al., 2003). Anxiety severity was measured with the Brief Symptom Inventory – Anxiety Subscale (Derogatis & Melisaratos, 1983). Previous antidepressant treatment adequacy was assessed using the Antidepressant Treatment History Form (Buchalter et al., 2019; Sackeim et al., 2019).

### Statistical analysis

All participants who completed at least 4 weeks of treatment and the Q-LES-Q at baseline and at least one post-treatment time point were included in this analysis. Given the overlapping age ranges in the two trials, participants from both trials were combined and divided into two groups: those aged 60 years or older were classified as ‘Older adults’, and those under 60 years as ‘Younger adults’. Older and younger adult groups were compared on baseline demographic and clinical variables using Kruskal–Wallis tests for continuous variables or chi-square tests for categorical variables. A mixed-effects repeated measures model explored whether age group (i.e. younger vs. older adults) was associated with change in QoL from baseline to end of treatment or to 12-week follow-up. Age group was set as the between-subject factor, the three time points were set as within-subjects factors, and participant was set as a random factor to account for missing data. Covariates included baseline Q-LES-Q score, baseline QIDS score, and treatment modality (i.e. one of the four rTMS modalities: unilateral high-frequency rTMS, unilateral iTBS, bilateral high/low-frequency rTMS, and bilateral iTBS/cTBS). A series of sensitivity analyses reported in detail in the Supplementary Materials was conducted to assess the robustness of findings, including a model with additional covariates (episode length, treatment history, education, and baseline anxiety), stratification by stimulation type (standard rTMS vs. TBS), within-trial analyses (THREE-D vs. FOUR-D), and propensity score-matched analyses balancing baseline QoL and depression severity. Next, change scores from baseline to end of treatment and to 12-week follow-up were calculated and compared between the younger and older age groups using Kruskal–Wallis tests. Effect sizes of changes were calculated for each age group and were classified as small (≤0.2), medium (>0.2 to <0.8), and large (≥0.8) (Cohen, 1988). Effect sizes were also calculated for changes in depression severity (i.e. QIDS) as a comparison. To further evaluate the clinical importance of the change in Q-LES-Q, we compared the proportion of participants with changes in Q-LES-Q above the previously established MCID of a 12-point change in younger and older adults using chi-square tests.

We used Spearman’s rank correlation coefficients to assess the correlation between Q-LES-Q scores and QIDS total scores at all time points, as well as between the changes in these scores from baseline to the end of treatment and 12-week follow-up.

Lastly, using the community norms of the Q-LES-Q described above, participants were classified into ‘normal QoL’, ‘mild to moderate QoL impairment’, and ‘severely impaired QoL’ at baseline, end of treatment, and 12-week follow-up. Proportions within the norm groups were compared between older and younger individuals using chi-square tests. Changes in proportions of participants in each norm group from baseline to the end of treatment or 12-week follow-up were compared using McNemar’s test. R Studio (version 4.1.2) was used to perform all analyses.

## Results

**Figure 1.**
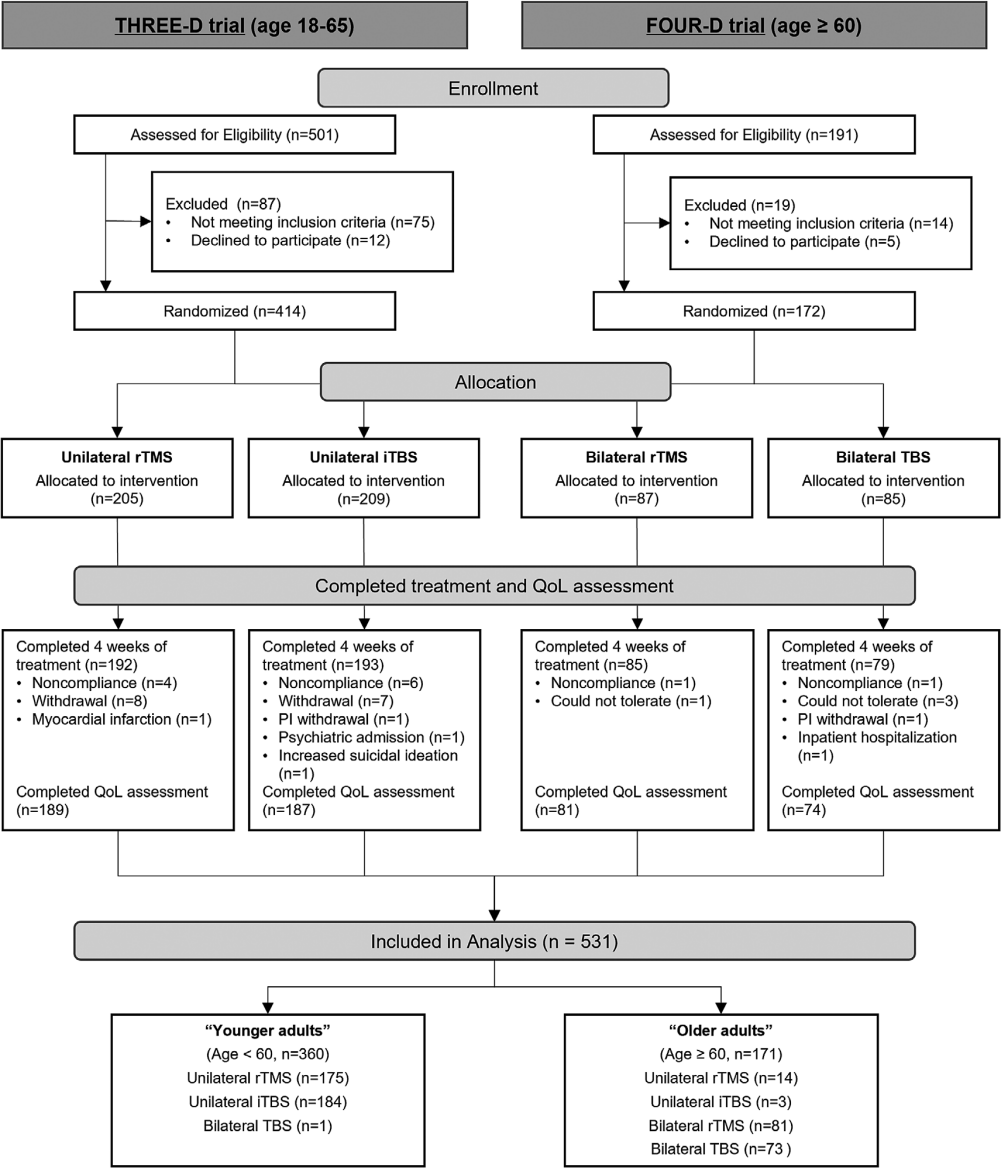
Flow diagram.

In total, 586 participants were enrolled, with 414 in the THREE-D trial and 172 in the FOUR-D trial. Of these, 531 (376 in THREE-D and 155 in FOUR-D) completed at least 4 weeks of treatment and the QoL assessment (Figure 1). Demographic and clinical characteristics were similar between included and excluded participants, except for slightly higher baseline anxiety severity in noncompleters (Supplementary Table 1). Participants were divided into two groups: 360 age < 60 years (‘Younger Adults’) and 171 age ≥ 60 years (‘Older Adults’) (Figure 1). Compared to younger adults, the older adults had significantly fewer education years, longer depressive episodes, more previous antidepressant treatment trials, less severe depressive symptoms, and higher baseline Q-LES-Q scores (Table 1).

**Table 1.** Baseline characteristics of the younger and older adult groups with depression

		Younger adults (age < 60 years) (*n* = 360)	Older adults (age ≥ 60 years) (*n* = 171)	*p*-value
Age, mean (SD)		41.7 (10.9)	66.1 (5.88)	<0.001
Sex, *n* (%)	*Male*	147 (40.8%)	74 (43.3%)	0.661
	*Female*	213 (59.2%)	97 (56.7%)	
Years of education, mean (SD)		16.3 (3.19)	15.5 (2.66)	0.001
Number of medication trials, mean (SD)		1.62 (0.88)	2.12 (1.41)	<0.001
Episode length, mean (SD)		22.6 (25.8)	56.7 (87.7)	<0.001
Age at onset, mean (SD)		21.0 (11.0)	30.6 (18.9)	<0.001
HRSD score, mean (SD)		23.6 (4.35)	18.9 (4.62)	<0.001
QIDS score, mean (SD)		17.0 (3.93)	15.9 (4.43)	0.014
BSI-A score, mean (SD)		10.1 (5.40)	10.1 (5.37)	0.955
Q-LES-Q score, mean (SD)		33.0 (12.8)	39.6 (14.7)	<0.001

*Note*: BSI-A, Brief Symptom Inventory–Anxiety; HRSD, 17-item Hamilton Rating Scale for Depression; QIDS, Quick Inventory of Depressive Symptomatology (16-item) (self-report); Q-LES-Q, Quality of Life Enjoyment and Satisfaction Questionnaire – Short Form (Q-LES-Q-SF) (percentage of the maximum score).

Comparisons were performed using Kruskal–Wallis *H* tests for continuous variables and *χ* ^2^ tests for categorical variables.

### QoL improvements after rTMS treatment

A mixed-effects repeated-measures model for Q-LES-Q score revealed a significant effect across the three time points (*F*(2, 983.22) = 175.70, *p* < .001), a significant effect of the baseline Q-LES-Q score (*F*(1, 541.72) = 294.46, *p* < .001), and a significant effect of baseline QIDS score (*F*(1, 551.94) = 5.59, *p* = .018). There was no significant main effect for age group (*F*(1, 528.05) = 1.61, *p* = .204) or treatment modality (*F*(3, 538.95) = 1.63, *p* = .182), and no significant interaction between age group and time point (*F*(2, 983.58) = 1.85, *p* = .158).

From baseline to end of treatment, the younger adult group experienced a mean (SD) increase of 14.7 (18.0) points with a large effect size (Cohen’s *d* = 0.81; 95% confidence interval [CI] = [0.69, 0.94]), while the older adult group experienced a mean (SD) increase of 12.2 (17.2) points with a medium effect size (Cohen’s *d* = 0.71; 95% CI = [0.54, 0.88]). These changes did not differ significantly between younger and older adult age groups (Table 2). An MCID on the Q-LES-Q from baseline to end of treatment was observed in 174/ 354 (49.2%) of younger adults and 73/170 (42.9%) of older adults, with no significant difference between these two percentages (*χ^2^*(1) = 1.54, *p* = .215).

**Table 2.** Q-LES-Q scores and norm groups at baseline, end of treatment, and 12-week follow-up

	All (age 18–89 years)	Younger adults (age < 60 years)	Older adults (age ≥ 60 years)	*p*-value
Q-LES-Q baseline				
*N*	531	360	171	
Mean score (SD)	35.1 (13.78)	33.0 (12.8)	39.6 (14.7)	<0.001
Norm group				0.001
Within normal QoL, *n* (%)	5 (0.9%)	1 (0.3%)	4 (2.3%)	
Mild to moderate QoL impairment, *n* (%)	28 (5.3%)	12 (3.3%)	16 (9.4%)	
Severely impaired QoL, *n* (%)	498 (93.8%)	347 (96.4%)	151 (88.3%)	
Q-LES-Q end of treatment				
* N*	524	354	170	
Mean score (SD)	49.1 (19.0)	47.7 (19.0)	51.8 (18.7)	0.021
Mean change from Baseline (SD)	13.9 (17.8)	14.7 (18.0)	12.2 (17.2)	0.140
Norm group				0.038
Within normal QoL, *n* (%)	103 (19.7%)	70 (19.8%)	33 (19.4%)	
Mild to moderate QoL impairment, *n* (%)	89 (17.0%)	50 (14.1%)	39 (22.9%)	
Severely impaired QoL, *n* (%)	332 (63.4%)	234 (66.1%)	98 (57.6%)	
Q-LES-Q 12-week follow-up				
* N*	387	249	138	
Mean score (SD)	51.1 (20.4)	49.6 (20.8)	53.8 (19.3)	0.057
Mean change from baseline (SD)	14.8 (19.6)	15.9 (20.2)	12.9 (18.5)	0.220
Norm group				0.324
Within normal QoL, *n* (%)	96 (24.8%)	59 (23.7%)	37 (26.8%)	
Mild to moderate QoL impairment, *n* (%)	62 (16.0%)	36 (14.5%)	26 (18.8%)	
Severely impaired QoL, *n* (%)	229 (59.2%)	154 (61.8%)	75 (54.3%)	

*Note*: Q-LES-Q, Quality of Life Enjoyment and Satisfaction Questionnaire – Short Form (Q-LES-Q-SF) (percentage of the maximum score).

*Note*: ‘Within normal QoL’ defined as Q-LES-Q score ≥ 67; ‘Mild to moderate QoL impairment’ defined as Q-LES-Q score 55.7–67; ‘Severely impaired QoL’ defined as Q-LES-Q score ≤ 55.7.

Comparisons were performed using Kruskal–Wallis *H* tests for continuous variables and *χ* ^2^ tests for categorical variables.

**Figure 2.**
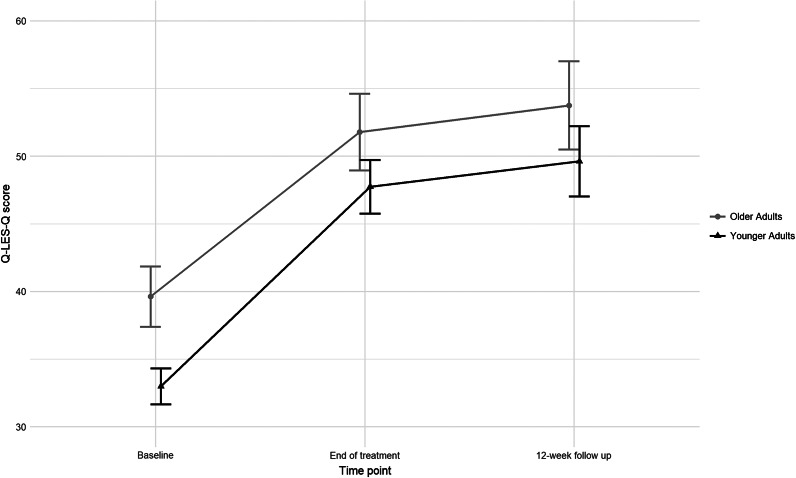
Changes in Q-LES-Q score from baseline to end of treatment and to 12-week follow-up for the younger and older adult groups with depression. Error bars represent confidence intervals.

From baseline to the 12-week follow-up, the younger adult group experienced a mean (SD) increase of 15.9 (20.2) points with a medium effect size (Cohen’s *d* = 0.79; 95% CI = [0.64, 0.93]), while the older adult group experienced a mean (SD) increase of 12.9 (18.5) points with a medium effect size (Cohen’s *d* = 0.70; 95% CI = [0.51, 0.88]) (Figure 2). These changes did not differ significantly between the younger and older adult groups (Table 2). An MCID on the Q-LES-Q from baseline to the 12-week follow-up was observed in 129/249 (51.8%) of younger and 66/138 (47.8%) of older adults, with no significant difference between the groups (*χ^2^*(1) = 0.41, *p* = .519).

### Association between QoL and depression severity

QIDS scores improved from baseline to end of treatment with a large effect size for both the younger (Cohen’s *d* = 1.16; 95% CI = [1.02, 1.29]) and older adult age groups (Cohen’s *d* = 1.01; 95% CI = [0.82, 1.19]). Similarly, large effect sizes were found from baseline to the 12-week follow-up for younger (Cohen’s *d* = 0.92; 95% CI = [0.76, 1.07]) and older adults (Cohen’s *d* = 0.91; 95% CI = [0.71, 1.11]). Moderate to strong correlations were observed between Q-LES-Q and QIDS scores at baseline (*r*_s_ = −0.61, *p* < .001), at the end of treatment (*r*_s_ = −0.79, *p* < .001), and at the 12-week follow-up (*r*_s_ = −0.83, *p* < .001). Strong, significant correlations were also found between changes in Q-LES-Q score and QIDS from baseline to end of treatment (*r*_s_ = −0.73, *p* < .001) and 12-week follow-up (*r*_s_ = −0.79, *p* < .001).

### QoL relative to community norms

At baseline, most participants were classified as having severely impaired QoL, with a significantly higher proportion in the younger adult age group (347/360, 96.4%) than in the older adult group (151/171, 88.3%), *χ^2^*(2) = 15.21, *p* < .001. The proportion of younger adults with severely impaired QoL significantly decreased to 66.1% (234/354) by the end of treatment (McNemar’s *χ* ^2^(3) = 111.09, *p* < .001), and to 61.8% (154/249) by the 12-week follow-up (McNemar’s *χ* ^2^(3) = 87.43, *p* < .001). Similarly, the proportion of older adults with severely impaired QoL significantly decreased to 57.6% (98/170) by the end of treatment (McNemar’s *χ* ^2^(3) = 51.13, *p* < .001) and to 54.3% (75/138) by the 12-week follow-up (McNemar’s *χ* ^2^(3) = 41.84, *p* < .001). Conversely, the proportion of younger adults classified as within-normal QoL significantly increased from 0.3% (1/360) at baseline to 19.8% (70/354) at the end of treatment, and to 23.7% (59/249) at the 12-week follow-up. Similarly, the proportion of older adults classified as within-normal QoL significantly increased from 2.3% (4/171) at baseline to 19.4% (33/170) at the end of treatment, and 26.8% (37/138) at the 12-week follow-up (Table 2).

## Discussion

We evaluated the effects of rTMS on self-reported QoL in both younger and older adults with depression using data from two non-inferiority trials on standard rTMS vs TBS (THREE-D and FOUR-D). To our knowledge, our study is one of the largest reporting QoL outcomes after rTMS and the first to examine potential differences between older and younger adults with depression. Our study has several findings. First, the older adult group had a significantly higher QoL at baseline than the younger adult group. Second, there were statistically significant and clinically meaningful improvements in QoL after rTMS, with no significant differences between younger and older participants. Third, there were strong and significant correlations between the improvements in self-reported QoL and self-reported depressive symptom severity. Lastly, the majority of participants exhibited severely impaired QoL at baseline, which improved significantly by the end of treatment and was sustained at the 12-week follow-up.

At baseline, we observed significantly better QoL and a slightly lower proportion of participants with severely impaired QoL in older participants than in younger participants (88.3% vs. 96.4%). Older participants also exhibited less severe depressive symptoms at baseline, which were moderately but significantly correlated with QoL. A similar pattern was also observed in an analysis from the STAR*D sample, where adults ≥65 reported better QoL and lower depressive symptom severity at baseline than adults <65 years (Steiner et al., 2017). Taken together, these findings suggest that younger adults are at greater risk of severe QoL impairment, possibly due to the more severe depressive symptoms observed in this age group. The observed differences in depressive symptom severity in the current study may also reflect the differences in inclusion criteria between the THREE-D and FOUR-D trials. Specifically, THREE-D required an HRSD-17 score ≥ 18, while FOUR-D required a MADRS score ≥ 18, which corresponds to an HRSD-17 score of 14 (Heo et al., 2007). This difference accounts for the more severely depressed younger participants than older participants in our sample.

With only 0.3% of the younger adult and 2.3% of the older adult group reporting ‘within-normal’ QoL at baseline, treatment of MDD should extend beyond clinical symptom management and encompass a focus on improving QoL. We observed significant improvements in QoL from baseline to end of treatment and to the 12-week follow-up, with medium to large effect sizes. These findings are consistent with previous studies demonstrating QoL improvements in younger adults following rTMS (Dumas et al., 2012; Giacobbe et al., 2020; Solvason et al., 2014). Our findings also extend these previous findings to older adults, showing that they experience similar benefits from rTMS. We found no significant differences in QoL changes between older and younger adults at either end of treatment or 12-week follow-up, and there was no significant interaction between age group and time point, suggesting that rTMS yields similar QoL benefits for both age groups.

We found slightly different effect sizes, with medium to large effect sizes in our younger adult group and medium effect sizes in the older adult group. This small difference may be explained by the higher baseline QoL in the older adult group, making improvements appear less pronounced relative to the younger adult group. A similar pattern was reported in the STAR*D sample, where adults ≥65 started with better QoL than those <65 and showed slightly smaller improvement in QoL following citalopram monotherapy, as indicated by smaller effect sizes (Steiner et al., 2017). Importantly, in our sensitivity analysis, matching 171 younger adults to the group of 171 older adults on baseline depression severity and baseline QoL again showed no significant differences in QoL improvement between age groups with nearly identical effect sizes across younger and older adult groups (see Supplementary Materials). This suggests that the small effect size differences observed in the unmatched sample were likely attributable to baseline group differences in QoL.

The improvement in QoL was clinically meaningful in both age groups, as 49.2% of the younger adult group and 42.9% of the older adult group exceeded the threshold for an MCID of a 12-point change on the Q-LES-Q. This threshold corresponds with the assessment of at least ‘minimally improved’ on the Clinical Global Impression-Improvement scale (i.e. a score of 1–3) (Endicott et al., 2007), and has been used in previous studies (Conway et al., 2018; Rush et al., 2021). Moreover, Cohen’s *d* effect sizes ranging from 0.79 to 0.81 for the younger adult group and from 0.70 to 0.71 for the older adult group are comparable or higher to those reported in previous studies. For instance, in the STAR*D trial, the level 1 treatment, citalopram, yielded effect sizes of 0.79 for adults <65 and 0.71 for adults ≥65 on the Q-LES-Q (Steiner et al., 2017). However, the majority of participants in our sample had one or more prior failed treatment trials, making it comparable to levels 2–4 treatment in the STAR*D trial – that is, switching antidepressant, augmenting them, or combining them with CBT – which yielded more modest effect sizes ranging from 0.20 to 0.52 (IsHak et al., 2015). Notably, 96.4% of younger participants and 88.3% of older participants in the current sample reported severely impaired QoL at baseline. By the end of treatment, these rates decreased to 66.1% for younger participants and 57.6% for older participants, further decreasing at the 12-week follow-up to 61.8 and 54.3%, respectively. For comparison, in the STAR*D levels 2–4, between 83.3 and 91.1% of participants reported severely impaired QoL at baseline, which decreased to between 59.5 and 81.1% following treatment. Together, our and STAR*D results suggest that rTMS has the potential to improve QoL as effectively as, or even surpassing, conventional second- or third-line treatments in both younger and older adults.

Finally, while improvements in QoL and self-reported depressive symptom severity were strongly correlated, effect sizes for QoL were slightly lower than those for depression severity. Specifically, medium to large effect sizes were observed for QoL (Cohen’s *d* ranging from 0.70 to 0.81), while larger effect sizes were found for depression severity (ranging from 0.91 to 1.16). However, QoL continued to show improvement during the 12-week follow-up, a pattern generally not observed with depressive symptoms, suggesting that the timing of different outcomes is a critical factor when evaluating treatment effects.

This temporal pattern, together with the larger effect sizes observed for depressive symptoms relative to QoL and their strong correlation, supports the notion that QoL improvements are largely driven by preceding symptom reduction, although the relationship between QoL and depressive symptoms is likely bidirectional. From a neurobiological perspective, QoL improvement after rTMS has been linked to decreased perfusion in the precuneus, a default mode network hub involved in self-referential processing (Dumas et al., 2012). In addition, nonspecific treatment factors, such as regular clinical contact and the structured routine of attending daily rTMS sessions, may further enhance perceived improvements in QoL.

Several limitations should be acknowledged. First, this analysis draws data from two different clinical trials to maximize the sample size and include a broad range of ages (ages 18–89 years). While both trials were similar in their design, they used slightly different inclusion criteria, which resulted in the inclusion of participants with more severe depressive symptoms in our younger adult group than in our older adult group. Moreover, participants of the THREE-D trial and the FOUR-D trial received different forms of rTMS or TBS. To account for this, we included treatment modality as a covariate in the model, and it showed no statistically significant effect on QoL outcomes. Second, QoL was measured using a single scale, the Q-LES-Q. Different scales may emphasize different domains of QoL dysfunction, and the use of alternative measures, such as the 12-item Short-Form Health Survey, might emphasize other aspects of QoL (Daly et al., 2010). Third, the primary trials did not include sham-stimulation control groups, so the observed effects cannot be conclusively attributed to rTMS treatment.

In summary, we found that rTMS significantly improved QoL in adults with depression across the lifespan, with sustained benefits during a follow-up period. Both younger and older participants showed significant and clinically meaningful improvements in QoL, with no significant differences between the two age groups. rTMS improved QoL in both age groups at levels comparable to, or better than, what has been reported with pharmacotherapy. While the absence of a sham control group limits definitive causal inference, our findings add to the evidence demonstrating the positive effects of rTMS on depressive symptoms and recent trials comparing rTMS to switch or augmentation pharmacotherapy (Dalhuisen et al., 2024; Papakostas et al., 2024). This suggests that rTMS should be considered as an earlier intervention when treating depression (Kaster & Blumberger, 2024), as it provides a promising option for reducing depressive symptom severity and enhancing QoL in both younger and older adults with depression. Future sham-controlled and long-term studies examining QoL and related functional outcomes after rTMS are needed to confirm these effects and establish the durability of QoL benefits beyond 12 weeks.

## Supporting information

Göke et al. supplementary material Göke et al. supplementary material

## Supplementary material

The supplementary material for this article can be found at http://doi.org/10.1017/S0033291725102079.
