# Supplementary material for: The effects of rTMS on self-reported quality of life in younger and older adults with major depressive disorder
Source: Psychol Med. 2025 Oct 8;55:e303. doi: 10.1017/S0033291725102079 (PMC12527504; doi:10.1017/S0033291725102079)
Supplement: Göke et al. supplementary material [file S0033291725102079sup001.docx]

**Supplementary Table 1**

*Baseline characteristics for completers and non-completers*

|  |  | **Completer**  (*n*= 531) | **Non-completer**  (*n*=55) | *p*-value |
| --- | --- | --- | --- | --- |
| Age, mean (SD) |  | 49.6 (14.9) | 49.3 (16.7) | 0.965 |
| Sex, n (%) | *Male* | 221 (41.6%) | 27 (49.1%) | 0.355 |
|  | *Female* | 310 (58.4%) | 28 (50.9%) |  |
| Years of education, mean (SD) |  | 16.0 (3.05) | 15.8 (2.82) | 0.662 |
| Treatment Allocation | *Unilateral rTMS* | 189 (35.6%) | 16 (29.1%) | 0.420 |
|  | *Unilateral iTBS* | 187 (35.2%) | 22 (40.0%) |  |
|  | *Bilateral rTMS* | 81 (15.3%) | 6 (10.9%) |  |
|  | *Bilateral TBS* | 74 (13.9%) | 11 (20.0%) |  |
| Number of medication trials, mean (SD) |  | 1.78 (1.10) | 1.55 (0.94) | 0.187 |
| Episode length, mean (SD) |  | 33.6 (56.3) | 41.5 (86.8) | 0.434 |
| Age at onset, mean (SD) |  | 24.1 (14.7) | 23.3 (13.3) | 0.920 |
| HRSD score, mean (SD) |  | 22.1 (4.94) | 22.9 (5.53) | 0.312 |
| QIDS score, mean (SD) |  | 16.6 (4.13) | 17.2 (4.34) | 0.280 |
| BSI-A score, mean (SD) |  | 10.1 (5.39) | 11.8 (5.60) | **0.033** |
| Q-LES-Q score, mean (SD) |  | 35.1 (13.8) | 31.9 (14.4) | 0.135 |

Abbreviations: BSI-A, Brief Symptom Inventory–Anxiety; HRSD, 17-item Hamilton Rating Scale for Depression; QIDS, Quick Inventory of Depressive Symptomatology (16-item) (self-report); Q-LES-Q, Quality of Life Enjoyment and Satisfaction Questionnaire Short Form (Q-LES-Q-SF) (percentage of the maximum score).

Comparisons were performed using Kruskal-Wallis *H* tests for continuous variables and χ^2^ tests for categorical variables.

**Sensitivity Analyses**

To address heterogeneity in patient characteristics and stimulation protocols across the two included trials, we conducted a series of sensitivity analyses aimed at evaluating the robustness of the observed quality of life (QoL) improvements and examining potential differences in treatment protocols.

**1. Extended covariate model**

To further examine whether baseline clinical heterogeneity could account for observed results, we re-estimated the mixed-effects model adding episode length, number of prior failed antidepressant trials, years of education, and baseline anxiety severity (BSI-A), in addition to the prespecified covariates (baseline Q-LES-Q, baseline QIDS, treatment modality). Age group was set as the between-subject factor, the three time points were set as within-subjects factors, and participant was set as a random factor.

Results remained largely consistent with the primary model. There was a significant effect across the three time points (*F*(2, 985.60) = 45.12, *p* < .001), and a significant effect of baseline Q-LES-Q score (*F*(1, 541.19) = 270.78, *p* < .001). No significant main effect of age group (*F*(1, 526.42) = 1.28, *p* = .258), treatment modality (*F*(3, 538.50) = 1.15, *p* = .330), or interaction between age group and time point (*F*(2, 983.36) = 1.85, *p* = .158) was observed.

While baseline QIDS score did not reach statistical significance (*F*(1, 549.89) = 3.15, *p* = .076) in this model, there was a significant main effect of baseline BSI-A (*F*(1, 569.74 = 4.13, p = .043), but no interaction between baseline BSI-A and time point (*F*(2, 998.21 = 0.05, *p* = .948), indicating that anxiety did not influence the trajectory of QoL improvements. Main effects of episode length, number of prior failed antidepressant trials, and years of education were non-significant (all *p* > .05).

**2. Stratification by Stimulation type (rTMS vs TBS)**

We next examined whether stimulation type influenced QoL outcomes. Stratifying by all four specific protocols (unilateral 10Hz rTMS, unilateral iTBS, bilateral 1Hz/10Hz rTMS, bilateral cTBS/iTBS) was not feasible, as treatment type was fully confounded with age group: While younger adults (THREE-D trial) only received unilateral protocols, most older adults (FOUR-D trial) received bilateral protocols. There is no consensus in the literature that bilateral stimulation is superior to unilateral stimulation suggesting that the effectiveness of both interventions is similar. As such, we stratified participants into two broader categories: standard rTMS (unilateral 10Hz or bilateral 1Hz/10Hz) and TBS (unilateral iTBS or bilateral cTBS/iTBS) and repeated the main analysis.

- **Standard rTMS (n=270):** QoL improved significantly over time (*F*(2, 499.33) = 93.31, *p* < .001). There were significant main effects of baseline Q-LES-Q score (*F*(1, 274.41) =115.62, *p* < .001) and baseline QIDS score (*F*(1, 283.75) = 14.40, *p* < .001), but no significant effects of age group (*F*(1, 270.68) = 0.35, *p* = .555), treatment protocol (bilateral vs unilateral; *F*(1, 270.18) = 1.68, *p* = .196), or age group by time point interaction (*F*(2, 499.40) = 2.42, *p* = .090).
- **TBS (n=261):** QoL also improved significantly over time (*F*(2, 475.05) = 82.50, *p* < .001). There was a significant main effect of baseline Q-LES-Q score (*F*(1, 257.05) = 174.99, *p* < .001), but baseline QIDS (*F*(1, 257.73) = 0.15, *p* = .698), age group (*F*(1, 237.56) = 0.84, *p* = .360), treatment protocol (bilateral vs unilateral; *F*(1, 237.32) = 0.57, *p* = .449), and age group by time point interaction (*F*(2, 475.71) = 0.27, *p* = .760) were non-significant.

These results suggest that across both standard rTMS and TBS, QoL improved robustly over time, and results were not significantly influenced by age group or whether stimulation was unilateral vs bilateral.

**3. Subgroup analysis by trial (THREE-D vs FOUR-D)**

To test whether age may predict QoL improvements within each trial, we examined each trial separately, thereby holding the stimulation approach constant (unilateral in THREE-D, bilateral in FOUR-D). Linear regression models were conducted, with the change in Q-LES-Q from baseline to end of treatment as the dependent variable. Predictors included age (continuous), baseline Q-LES-Q, baseline QIDS, and treatment type (standard rTMS vs TBS).

- **THREE-D trial (ages 18–65, unilateral stimulation, n = 376):** Older age (β = 0.20, 95% CI [-0.04, 0.36], *p* = .013) and lower baseline Q-LES-Q (β = -0.42, 95% CI [-0.58, -0.25], *p* < .001) predicted greater improvement. Baseline QIDS and treatment type were not significant predictors (all *p* > .05).
- **FOUR-D trial (ages ≥ 60, bilateral stimulation, n = 155):** Lower baseline Q-LES-Q (β = -0.57, 95% CI [-0.81, -0.34], *p* < .001) and lower baseline QIDS (β = -0.84, 95% CI [-1.85, -0.10], *p* = .026) both significantly predicted greater QoL improvements. Neither age nor treatment type predicted change in QoL (*p* > .05).

These results suggests that while QoL improvements were similar between the younger and older adult groups, older age was modestly associated with greater QoL improvements within the younger adult cohort (THREE-D, unilateral stimulation), but not among older adults (FOUR-D, bilateral stimulation).

**4. Matched-Sample Analysis**

Because the younger and older adult groups differed significantly at baseline in both QoL and depression severity, we conducted a 1:1 propensity score matching. A subgroup of 171 younger adults was matched to the 171 older adults on baseline Q-LES-Q and QIDS scores, resulting in balanced groups on these variables (Q-LES-Q: 38.5 ± 13.1 vs 39.6 ± 14.7, *p* = .448; QIDS: 16.0 ± 3.8 vs 15.9 ± 4.4, *p* = .804).

In line with the primary analysis, QoL improved significantly over time (*F*(2, 644.77) = 117.69, *p* < .001), with significant main effects baseline Q-LES-Q (*F*(1, 345.28) = 214.22, *p* < .001) and baseline QIDS (*F*(1, 350.02) = 5.05, *p* = .025). There was no main effect of age group (*F*(1, 343.56) = 1.48, *p* = .224) or treatment modality (*F*(3, 348.29) = 1.67, *p* = .172), and no age group by time point interaction (*F*(2, 645.06) = 0.34, *p* = .713).

From baseline to end of treatment, the matched younger adult group experienced a mean (SD) increase of 12.5 (17.1) points with a medium effect size (*Cohen's d* = 0.73; 95% CI [0.56, 0.90]), while the older adult group experienced a mean (SD) increase of 12.2 (17.2) points with a medium effect size (*Cohen's d* = 0.71; 95% CI [0.54, 0.88]). From baseline to the 12-week follow-up, the matched younger adult group experienced a mean (SD) increase of 14.5 (20.5) points with a medium effect size (*Cohen's d* = 0.71; 95% CI [0.51–0.90]), while the older adult group experienced a mean (SD) increase of 12.9 (18.5) points with a medium effect size (*Cohen's d* = 0.70; 95% CI [0.51, 0.88]).

These results suggest that when baseline differences were minimized through matching, younger and older adults demonstrated highly similar improvements in QoL with nearly identical effect sizes both at the end of treatment and the 12-week follow-up.
